# Isolated hepatic perfusion as a treatment for uveal melanoma liver metastases (the SCANDIUM trial): study protocol for a randomized controlled trial

**DOI:** 10.1186/1745-6215-15-317

**Published:** 2014-08-09

**Authors:** Roger Olofsson, Lars Ny, Malin Sternby Eilard, Magnus Rizell, Christian Cahlin, Ulrika Stierner, Ulf Lönn, Johan Hansson, Ingrid Ljuslinder, Lotta Lundgren, Gustav Ullenhag, Jens Folke Kiilgaard, Jonas Nilsson, Per Lindnér

**Affiliations:** Department of Surgery, Institute of Clinical Sciences, Sahlgrenska Academy at University of Gothenburg, Sahlgrenska University Hospital, Blå stråket 5, 413 45 Gothenburg, Sweden; Department of Oncology, Institute of Clinical Sciences, Sahlgrenska Academy at University of Gothenburg, Sahlgrenska University Hospital, Blå stråket 5, 413 45 Gothenburg, Sweden; Transplant Institute, Institute of Clinical Sciences, Sahlgrenska Academy at University of Gothenburg, Sahlgrenska University Hospital, 413 45 Gothenburg, Sweden; Department of Oncology, Linköping University Hospital, Garnisonsvägen 10, 581 85 Linköping, Sweden; Department of Oncology, Karolinska University Hospital, Karolinska vägen, 171 76 Stockholm, Sweden; Department of Oncology, Norrlands University Hospital, 901 85 Umeå, Sweden; Department of Oncology, Skåne University Hospital, Getingevägen 4, 221 85 Lund, Sweden; Department of Radiology, Oncology and Radiation Science, Section of Oncology, Uppsala University, 751 05 Uppsala, Sweden; Department of Ophthalmology, Glostrup Hospital, Copenhagen University Hospital Glostrup, Nordre Ringvej 57, 2600 Glostrup, Denmark; Sahlgrenska Cancer Center, Sahlgrenska Academy at University of Gothenburg, Sahlgrenska University Hospital, Medicinaregatan 1F, 405 30 Gothenburg, Sweden

**Keywords:** Uveal melanoma, Liver metastases, Isolated hepatic perfusion, Regional treatment

## Abstract

**Background:**

Uveal melanoma is the most common primary intraocular malignancy in adults. Despite successful control of the primary tumor, metastatic disease will ultimately develop in approximately 50% of patients, with the liver being the most common site for metastases. The median survival for patients with liver metastases is between 6 and 12 months, and no treatment has in randomized trials ever been shown to prolong survival. A previous phase II trial using isolated hepatic perfusion (IHP) has suggested a 14-month increase in overall survival compared with a historic control group consisting of the longest surviving patients in Sweden during the same time period (26 versus 12 months).

**Methods/Design:**

This is the protocol for a multicenter phase III trial randomizing patients with isolated liver metastases of uveal melanoma to IHP or best alternative care (BAC). Inclusion criteria include liver metastases (verified by biopsy) and no evidence of extra-hepatic tumor manifestations by positron emission tomography–computed tomography (PET-CT). The primary endpoint is overall survival at 24 months, with secondary endpoints including response rate, progression-free survival, and quality of life. The planned sample size is 78 patients throughout five years.

**Discussion:**

Patients with isolated liver metastases of uveal melanoma origin have a short expected survival and no standard treatment option exists. This is the first randomized clinical trial to evaluate IHP as a treatment option with overall survival being the primary endpoint.

**Trial registration:**

ClinicalTrials.gov registration number: NCT01785316 (registered 1 February 2013). EudraCT registration number: 2013-000564-29.

## Background

Uveal melanoma is the most common primary intraocular malignancy in adults. Despite successful control of the primary tumor, patients with uveal melanoma have a high risk of developing metastatic disease. In the Collaborative Ocular Melanoma Study (COMS), the cumulative five and 10-year rates were 25% and 34% respectively [1]. Moreover, late metastases are frequent, with approximately 50% of the patients ultimately developing metastases [2]. The liver is the most common site for metastases and about 50% of the patients with advanced disease will have isolated liver metastases. These metastases are generally refractory to systemic chemotherapy and the median survival for patients with liver metastases is about six months. Regardless of treatment, the mortality rate is approximately 90% at two years with only about 1% of patients surviving more than five years [1].

Conventional systemic chemotherapy has failed to show any prolonged survival. Dacarbazine and temozolomide [3], as well as different multidrug regimens such as gemcitabine together with treosulfan [4], or the BOLD regimen (bleomycin, vincristine, lomustine, and dacarbazine) [5], have reported response rates of less than 10%. There are also reports of newer drugs which have proven efficacy in cutaneous malignant melanoma. At the 2013 American Society of Clinical Oncology (ASCO) meeting a randomized trial comparing the mitogen-activated protein kinase kinase (MEK) inhibitor selumetinib and temozolomide was reported. The trial included 80 patients and the results showed a response rate of 15% for selumetinib and a significant increase in progression-free survival (PFS) in nine weeks [6]. Two small trials have been published evaluating the efficacy and safety of ipilimumab in the treatment of metastatic uveal melanoma. The response rates have been low, with one partial response (PR) and three stable disease (SD) in a total of 35 patients. The median survival was reported to be nine months and five months respectively in the two trials [7, 8]. Currently, there are several phase I and II clinical trials evaluating the potential effects of new targeted therapies, either in monotherapy or in different combinations. The trials include trametinib (MEK-inhibitor), AEB001 (protein kinase C inhibitor), BVD-523 (extracellular signal-related kinase (ERK) inhibitor), and GSK2141795 (protein kinase B (AKT) inhibitor).

For patients with isolated liver metastases several different loco-regional liver treatment strategies have been explored. A retrospective study by Mariani *et al*. analyzed 3873 patients with uveal melanoma, of these 798 developed liver metastases, and 255 patients later underwent surgical resection with an overall survival (OS) of 14 months [9]. In the highly selected group of 76 patients where a R0 resection was achieved, a median overall survival of 27 months was reported. The European Organisation for Research and Treatment of Cancer (EORTC) 18021 study compared hepatic intra-arterial chemotherapy (HIA) with systemic chemotherapy and showed a modest increase in overall response rate for HIA (12 versus 2%), but no significant improvement in OS [10]. Transarterial chemoembolization (TACE) consists of a hepatic artery embolization with a simultaneous infusion of concentrated doses of a chemotherapeutic drug. Huppert *et al*. reported the results of TACE with cisplatin or carboplatin in 14 patients with uveal melanoma and liver metastases. Eight patients (57%) achieved a PR, four patients (29%) were assessed as SD, and two patients (14%) experienced tumor progression. Median time to progression was nine months and median survival was 12 months [11]. Selective internal radiation therapy (SIRT), mostly using the beta-emitting isotope Yttrium-90, has been used to deliver high-dose radiation to hepatic metastases. In a study by Klingenstein *et al*., 13 patients underwent SIRT, resulting in PR in eight patients (62%) and SD in two patients (15%) with a median survival time of seven months [12].

Isolated hepatic perfusion (IHP) is a regional treatment that was first performed more than 40 years ago [13]. During IHP, the liver is completely isolated from the systemic circulation, allowing a high concentration of chemotherapy to be perfused through the liver with minimal systemic exposure. In a previous study from our institution, improvements in the IHP procedure resulted in, minimized morbidity and mortality over time [14]. A phase II follow-up study confirms that IHP is a promising technique with tolerable morbidity [15]. There are currently no randomized trials using IHP, but in an attempt to find out whether the treatment prolongs OS we did a register study. The study showed a 14-month increased survival (26 versus 12 months) when comparing patients treated with IHP with the longest surviving patients in Sweden during the same time period [15].

In the early 1990s, three independent groups developed the percutaneous hepatic perfusion (PHP) technique combining a conventional hepatic artery infusion with a dual-balloon vena cava catheter collecting the outflow from the liver. The venous outflow was then connected to an extracorporeal venous bypass circuit, including a carbon filter, to recover any of the drug that was not absorbed by the liver [16–18]. A phase III study randomized 93 patients to either PHP or best alternative care (BAC). There was a significant increase in the primary endpoint, hepatic PFS (245 versus 49 days), and also in the overall response (34 versus 2%). Any conclusions concerning OS in this study are difficult to make due to the high proportion of patients crossing over from BAC to PHP [19].

In summary, many different treatment strategies have been explored for patients with liver metastases from uveal melanoma, but no one strategy has yet been proven to yield a survival benefit. The aim of this randomized study is to evaluate if IHP increases OS compared with BAC in patients with isolated liver metastases from uveal melanoma.

## Methods/Design

This is a prospective multicenter trial planning to randomize 78 patients to either IHP or BAC in a 1:1 ratio. The inclusion and exclusion criteria are depicted in Table 1. Randomization will be performed using permutated blocks with variable size stratified for study site. The study was approved by the Regional Ethical Review Board at the University of Gothenburg (reference number: 144–13), and all patients will provide written informed consent before inclusion in the trial.

**Table 1:** **Inclusion and exclusion criteria**

Inclusion criteria	
1.	Male or female aged above 18 years.
2.	Signed and dated written informed consent before the start of specific protocol procedures.
3.	Liver metastases measurable by magnetic resonance imaging (MRI, preferred) or a computational tomography (CT) scan of thorax and abdomen according to RECIST version 1.1 with at least one unidimensional measurable lesion ≥ 10 mm. The examination should be within four weeks prior to randomization.
4.	ECOG performance status of 0 or 1.
5.	No previous chemotherapy, radiotherapy, or biologic therapy for uveal melanoma metastases (first-line therapy)
6.	Adequate hepatic function (defined as ASAT,ALAT, bilirubin < = 3*ULN and PK-INR < = 1.5) and no medical history of liver cirrhosis or portal hypertension
**Exclusion criteria**	
1.	More than 50% of the liver volume (measured by CT or MRI) replaced by tumor.
2.	Evidence of extrahepatic disease by PET-CT
3.	Life expectancy of less than four months
4.	Pregnant or breast-feeding. Women of childbearing potential must have a negative pregnancy test performed within seven days prior to the start of study.
5.	Active infection.
6.	Ischemic cardiac disease or history of congestive heart failure with an LVEF < 40%.
7.	COPD or other chronic pulmonary disease with PFT’s indicating an FEV < 50% predicted for age.
8.	Reduced renal function defined as s-creatinine > =1.5 × ULN or creatinine clearance < 40 mL/min, calculated using the Cockroft and Gault formula.
9.	Reduced blood leukocytes or platelets defined as LPK < 2.0 × 10^9^/L and TPK <100 × 10^9^/L
10.	Use of live vaccines four weeks before or after the start of study.
11.	Body mass index above 35.

### Inclusion and exclusion criteria

Inclusion criteria

1. Male or female aged above 18 years.
2. Signed and dated written informed consent before the start of specific protocol procedures.
3. Liver metastases measurable by magnetic resonance imaging (MRI, preferred) or a computational tomography (CT) scan of thorax and abdomen according to Response Evaluation Criteria in Solid Tumors (RECIST) criteria version 1.1 with at least one unidimensional measurable lesion ≥ 10 mm. The examination should be within four weeks prior to randomization.
4. Eastern Cooperative Oncology Group (ECOG) performance status of 0 or 1.
5. No previous chemotherapy, radiotherapy, or biologic therapy for uveal melanoma metastases (first-line therapy).
6. Adequate hepatic function (defined as aspartate aminotransferase (AST), alanine aminotransferase (ALT), bilirubin < = 3 times upper limit of normal (ULN) and prothrombin time international normalized ratio (PT-INR) < = 1.5) and no medical history of liver cirrhosis or portal hypertension.

Exclusion criteria

1. More than 50% of the liver volume (measured by CT or MRI) replaced by tumor.
2. Evidence of extrahepatic disease by Positron emission tomography–computed tomography (PET-CT).
3. Life expectancy of less than four months.
4. Pregnant or breast-feeding. Women of childbearing potential must have a negative pregnancy test performed within seven days prior to the start of study.
5. Active infection.
6. Ischemic cardiac disease or history of congestive heart failure with an Left Ventricular Ejection Fraction (LVEF) < 40%.
7. Chronic obstructive pulmonary disease (COPD) or other chronic pulmonary disease with pulmonary function tests indicating a forced expiratory volume (FEV) < 50% predicted for age.
8. Reduced renal function defined as s-creatinine > =1.5 × ULN or creatinine clearance < 40 mL/min, calculated using the Cockroft and Gault formula.
9. Reduced blood leukocytes or platelets defined as a leucocyte count < 2.0 × 10^9^/L and a platelet count <100 × 10^9^/L
10. Use of live vaccines four weeks before or after the start of study.
11. Body mass index above 35.

### Arm A: isolated hepatic perfusion (IHP)

Patients will be treated with IHP at Sahlgrenska University Hospital, Göteborg, Sweden. The procedure is performed under general anaesthesia. An L-shaped midline incision extended under the right hypochondrium is made and the intrahepatic tumor volume, as well as signs of extra hepatic spread, is evaluated. Wire reinforced catheters (Medtronic Inc, Minnesota, USA) are inserted into the iliac vein and the axillary vein and connected to an external centrifugal pump (Maquet, Jostra Medizintechnik AG, Hirrlingen, Germany) to allow for shunting of blood from the lower extremities during the procedure when the caval vein is clamped. The caval vein is isolated infrahepatically above the renal veins and suprahepatically between the liver and the diaphragm. The right adrenal vein is clamped or ligated. Using the right gonadal vein, a catheter is placed in the retrohepatic portion of the caval vein for perfusion outflow. The portal vein is clamped and the proper hepatic artery is cannulated via the gastroduodenal artery. The catheters are then connected to the perfusion system. The liver perfusion is performed at a flow rate of 500 to 1200 ml/min with a target liver temperature of 40°C. The temperatures are continuously registered with thermistor probes (Exacon Scientific A/S, Roskilde, Denmark) placed in the inflow catheter and in the liver parenchyma. The leakage from the system is continuously recorded using technetium labelled albumin (Vasculosis Cis Bio, Gif-Sur-Yvette, France) injected into the perfusion circuit and measured using scintillation detector placed over the veno-venous bypass pump. All measurements are recorded and stored via the MedicView (Systemdata, Gothenburg, Sweden) computer system. When steady-state conditions in the perfusion circuit are established, melphalan (Alkeran, Aspen Europe GmbH, Bad Oldesloe, Germany) (1 mg/kg bodyweight divided into two doses, given 30 minutes apart) is added to the perfusion system. The perfusion is then continued for 60 minutes, after which the perfusion is discontinued and the liver is irrigated with Ringer-Acetate (Ringer Acetat, Baxter, Sweden). The shunts and the perfusion circuit are disconnected and the catheters are removed. If the patients progress systemically or in the liver, they will be treated with BAC (crossover to treatment arm B) but will be analyzed as being randomized to IHP according to intention-to-treat principles.

### Arm B: best alternative care (BAC)

The treating physician at each study center will decide the treatment, together with the patient. All available treatments, including surgery and other experimental treatments are tolerated, however no crossover to arm A (IHP) will be allowed.

### Follow-up

Patients will be followed actively according to this protocol for two years at each study center. Study visits will be carried out at each center and performed at baseline and after 3, 6, 12, 18, and 24 months and will include a CT or MRI scan of the liver (same modality as baseline examination) and a European Quality of Life Five Dimension Three Level Scale (EQ-5D-3 L) quality of life questionnaire. Any further examinations are at the discretion of the treating doctor.

### Endpoints

The primary objective of the study is OS, defined as the frequency of individuals alive at 24 months.

Secondary objectives include:

Median OS, defined as time from randomization to death and analyzed using Kaplan-Meier and the log-rank test. Hepatic PFS, defined as time from randomization to progression of existing lesions, or appearance of new lesions, within the liver according to RECIST criteria (version 1.1) using a CT or MRI scan [20]. Response, defined as best response according to RECIST criteria (version 1.1) using a CT or MRI scan [20]. For the BAC group, best response during the whole follow-up period of 24 months, for the IHP group, best response until hepatic progression (the time point when crossover to the BAC group is allowed). Health economic evaluation, to be measured by quality-adjusted life year (QALY), which will be estimated using EQ-5D-3 L at baseline and at 3, 6, 12, 18, and 24 months. Any serious adverse event(SAE) or Suspected Unexpected Serious Adverse Reactions SUSAR that occurs in either of the two groups will be reported.

### Sample size calculation

It is expected that the percentage of subjects who reach the primary endpoint of OS after 24 months will be 50% in the study group and 20% in the control group. Based on this assumption, 78 subjects are planned to be randomized to the two treatment groups in a 1:1 ratio (IHP:BAC) to achieve 80% power for the superiority comparison (Fisher’s exact test) of the primary endpoint between the two treatment groups. This allows for a two-sided type I error of 5% and a 5% dropout rate. Enrolment will continue until the required sample size has been randomized; a 60-month recruitment time is expected.

### Data monitoring committee (DMC)

An independent Data Monitoring Committee (DMC) will be appointed and will consist of one physician and one statistician, neither with any other involvement in the study. For efficacy the DMC should use Ó’Brian-Flemming group sequential boundaries. The DMC should also look for safety and at conditional power when giving advice regarding the continuation of the study. The DMC should start assessing efficacy data after 40% of the subjects have completed the study.

### Biobanking

Tumor biopsies will be obtained during IHP and these samples will be used to genetically characterize the tumors by DNA and RNA sequencing, and to develop mouse xenograft models. We will also collect blood samples from all patients at different predefined time points and these samples will be analyzed for biomarkers using biased ELISA methods or unbiased proteomics approaches.

## Discussion

IHP using melphalan is a major surgical procedure with associated risks. This procedure was implemented in the 1980s at Sahlgrenska University Hospital, Gothenburg, Sweden. A study from our institution reports how the IHP procedure has improved by sequential changes in the treatment protocol. Before 2005 there was a high frequency of postoperative mortality, however after excluding patients with a high tumor burden and by lowering the melphalan dose to 1 mg/kg bodyweight, there has been no mortality in more than 50 patients treated since 2005 [14, 15].

Liver insufficiency due to IHP has predominantly been reported in patients with a high tumor burden (above 50% of the liver volume), and these patients are excluded from this study. This side effect is an acute toxicity and will be detected during the immediate postoperative period (within five days) by daily monitoring of liver function tests (LFTs).

The current morbidity related to the procedure has been attributed to complications of the surgical intervention itself, in particular postoperative infections and thromboembolic events. To reduce this risk, patients receive preoperative prophylaxis with antibiotics, as well as a 10-day prophylactic regimen of low-molecular weight heparin postoperatively. During the study, SAE will be reported for both treatment arms, except for events commonly related to cancer disease. For patients treated with IHP, all AE grade III to V will also be recorded. The DMC will perform safety analyses during the study and can recommend discontinued inclusion in the study to the steering group.

Taken together, there is a risk to undergoing IHP that could be compared to other major surgeries (such as liver resections) with associated complications and a small risk of postoperative mortality. However, for this group of patients with a short expected survival and no standard treatment option, the possible advantages of improved survival are considered to outweigh the risks.

This group of patients (with liver metastases of ocular melanoma origin) has a dismal prognosis and no proven therapy exists that prolongs survival. The median survival is between six and 12 months and there are very few patients that can be considered long-term survivors (three to five years). In a retrospective study IHP has shown a prolonged OS of 14 months (median) compared to the longest surviving patients in Sweden during the same time period [15]. A sample size calculation based on these data, and with a power of 80% to detect a superiority in the primary endpoint, has shown that 78 patients are required to participate in the study.

One weakness of the trial is the limited amount of patients included, but with a rare disease it is important to balance the effect size with the possibility of conducting the trial at all. IHP is a complex procedure, and a survival benefit of a few weeks or months is probably not clinically relevant, and this partly justifies the risk of a negative result even if there would be a small gain in survival not detected by an underpowered trial.

The fields of targeted therapies and immunotherapies are rapidly evolving and the expected recruitment period of the study is five years. To avoid the control regimen becoming obsolete (due to other competing trials) and recruitment being halted, we have decided that BAC (including other experimental treatments) will be the control regimen. As patients in the IHP arm are also allowed to receive BAC after progression, this study will evaluate if IHP adds survival time to the best alternative treatment available during the study period.

Uveal melanoma most frequently carry mutations in the *GNAQ*, *GNA11*, and *BAP1* genes, where GNAQ/GNA11 mutations are mutually exclusive [21–23]. With the genetic information obtained from the material in our IHP biobank, we can investigate if different mutations impact response rates or OS. It is possible, and even likely, that knowing the mutation status of the tumor sample would impact the treatment in arm B upon crossover. However, we do not believe that this means better systemic treatments for patients randomized to arm B, since it is anticipated that mutation analyses affecting treatment decisions will be undertaken for all patients in the study. RNA sequencing can also generate expression signatures that correlate with different survival outcomes. Another benefit of biobanking blood and tumor tissue is that new animal models of uveal melanoma can be developed, and that an increased knowledge concerning biomarkers of response can be acquired.

## Trial status

The study began enrolment in June 2013.

## Abbreviations

AE: Adverse event
BAC: Best alternative care
CR: Complete response
CT: Computed tomography
DMC: Data Monitoring Committee
HIA: Hepatic intra-arterial chemotherapy
IHP: Isolated hepatic perfusion
LFTs: Liver function test
MRI: Magnetic resonance imaging
OS: Overall survival
PD: Progressive disease
PET-CT: Positron emission tomography-computed tomography
PFS: Progression-free survival
PHP: Percutaneous hepatic perfusion
PR: Partial response
QALY: Quality-adjusted life years
RECIST: Response Evaluation Criteria In Solid Tumors
SAE: Serious adverse event
SD: Stable disease
SIRT: Selective internal radiation therapy
SUSAR: Suspected unexpected serious adverse reactions
TACE: Transarterial chemoembolization.

## References

[1] Diener-West M, Earle JD, Fine SL, Hawkins BS, Moy CS, Reynolds SM, Schachat AP, Straatsma BR (2001). The COMS randomized trial of iodine 125 brachytherapy for choroidal melanoma, III: initial mortality findings. COMS Report No. 18. Arch Ophthalmol.

[2] Kujala E, Makitie T, Kivela T (2003). Very long-term prognosis of patients with malignant uveal melanoma. Invest Ophthalmol Vis Sci.

[3] Bedikian AY, Papadopoulos N, Plager C, Eton O, Ring S (2003). Phase II evaluation of temozolomide in metastatic choroidal melanoma. Melanoma Res.

[4] Schmittel A, Scheulen ME, Bechrakis NE, Strumberg D, Baumgart J, Bornfeld N, Foerster MH, Thiel E, Keilholz U (2005). Phase II trial of cisplatin, gemcitabine and treosulfan in patients with metastatic uveal melanoma. Melanoma Res.

[5] Kivela T, Suciu S, Hansson J, Kruit WH, Vuoristo MS, Kloke O, Gore M, Hahka-Kemppinen M, Parvinen LM, Kumpulainen E, Humblet Y, Pyrhonen S (2003). Bleomycin, vincristine, lomustine and dacarbazine (BOLD) in combination with recombinant interferon alpha-2b for metastatic uveal melanoma. Eur J Cancer.

[6] Carvajal RD, Sosman JA, Quevedo F (2013). Phase II study of selumetinib (sel) versus temozolomide (TMZ) in gnaq/Gna11 (Gq/11) mutant (mut) uveal melanoma (UM). J Clin Oncol.

[7] Danielli R, Ridolfi R, Chiarion-Sileni V, Queirolo P, Testori A, Plummer R, Boitano M, Calabro L, Rossi CD, Giacomo AM, Ferrucci PF, Ridolfi L, Altomonte M, Miracco C, Balestrazzi A, Maio M (2012). Ipilimumab in pretreated patients with metastatic uveal melanoma: safety and clinical efficacy. Cancer Immunol Immunother.

[8] Kelderman S, van der Kooij MK, van den Eertwegh AJ, Soetekouw PM, Jansen RL, van den Brom RR, Hospers GA, Haanen JB, Kapiteijn E, Blank CU (2013). Ipilimumab in pretreated metastastic uveal melanoma patients. Results of the Dutch Working group on Immunotherapy of Oncology (WIN-O). Acta Oncol.

[9] Mariani P, Piperno-Neumann S, Servois V, Berry MG, Dorval T, Plancher C, Couturier J, Levy-Gabriel C, Lumbroso-Le Rouic L, Desjardins L, Salmon RJ (2009). Surgical management of liver metastases from uveal melanoma: 16 years’ experience at the institut curie. Eur J Surg Oncol.

[10] Leyvraz S, Suciu S, Piperno-Neumann S, Baurain J, Zdzienicki M, Testori A, Marshall E, Scheulen M, Jouary T, Negrier S, Vermorken J, Kaempgen E, Durando X, Schadendorf D, Gurunath R, Polders L, De Schaetzen G, Vanderschaeghe S, Gauthier M, Keilholz U (2012). Randomized phase III trial of intravenous (IV) versus hepatic intra-arterial (HIA) fotemustine in patients with liver metastases from uveal melanoma: Final results of the EORTC 18021 study. J Clin Oncol.

[11] Huppert PE, Fierlbeck G, Pereira P, Schanz S, Duda SH, Wietholtz H, Rozeik C, Claussen CD (2010). Transarterial chemoembolization of liver metastases in patients with uveal melanoma. Eur J Radiol.

[12] Klingenstein A, Haug AR, Zech CJ, Schaller UC (2013). Radioembolization as locoregional therapy of hepatic metastases in uveal melanoma patients. Cardiovasc Intervent Radiol.

[13] Ausman RK, Aust JB (1960). Isolated perfusion of the liver with HN2. Surg Forum.

[14] Rizell M, Mattson J, Cahlin C, Hafstrom L, Lindner P, Olausson M (2008). Isolated hepatic perfusion for liver metastases of malignant melanoma. Melanoma Res.

[15] Olofsson R, Cahlin C, All-Ericsson C, Hashimi F, Mattsson J, Rizell M, Lindner P (2014). Isolated hepatic perfusion for ocular melanoma metastasis: registry data suggests a survival benefit. Ann Surg Oncol.

[16] Ku Y, Saitoh M, Nishiyama H, Fujiwara S, Iwasaki T, Ohyanagi H, Saitoh Y (1989). Extracorporeal adriamycin-removal following hepatic artery infusion: use of direct hemoperfusion combined with veno-venous bypass. Nihon Geka Gakkai zasshi.

[17] Curley SA, Newman RA, Dougherty TB, Fuhrman GM, Stone DL, Mikolajek JA, Guercio S, Guercio A, Carrasco CH, Kuo MT (1994). Complete hepatic venous isolation and extracorporeal chemofiltration as treatment for human hepatocellular carcinoma: a phase I study. Ann Surg Oncol.

[18] Beheshti MV, Denny DF, Glickman MG, Bodden W, Marsh JC, Strair R, Ravikumar TS (1992). Percutaneous isolated liver perfusion for treatment of hepatic malignancy: preliminary report. J Vasc Interv Radiol.

[19] Pingpank JF, Hughes MS, Alexander HR, Faries MB, Zager JS, Royal R, Whitman ED, Nutting CW, Siskin GP, Agarwala SS (2010). A phase III random assignment trial comparing percutaneous hepatic perfusion with melphalan (PHP-mel) to standard of care for patients with hepatic metastases from metastatic ocular or cutaneous melanoma. J Clin Oncol.

[20] Eisenhauer EA, Therasse P, Bogaerts J, Schwartz LH, Sargent D, Ford R, Dancey J, Arbuck S, Gwyther S, Mooney M, Rubinstein L, Shankar L, Dodd L, Kaplan R, Lacombe D, Verweij J (2009). New response evaluation criteria in solid tumours: Revised RECIST guideline (version 1.1). Eur J Cancer.

[21] Van Raamsdonk CD, Bezrookove V, Green G, Bauer J, Gaugler L, O’Brien JM, Simpson EM, Barsh GS, Bastian BC (2009). Frequent somatic mutations of GNAQ in uveal melanoma and blue naevi. Nature.

[22] Van Raamsdonk CD, Griewank KG, Crosby MB, Garrido MC, Vemula S, Wiesner T, Obenauf AC, Wackernagel W, Green G, Bouvier N, Sozen MM, Baimukanova G, Roy R, Heguy A, Dolgalev I, Khanin R, Busam K, Speicher MR, O'Brien J, Bastian BC (2010). Mutations in GNA11 in uveal melanoma. N Engl J Med.

[23] Harbour JW, Onken MD, Roberson ED, Duan S, Cao L, Worley LA, Council ML, Matatall KA, Helms C, Bowcock AM (2010). Frequent mutation of BAP1 in metastasizing uveal melanomas. Science.

