# Erratum to: Isolated hepatic perfusion as a treatment for uveal melanoma liver metastases (the SCANDIUM trial): study protocol for a randomized controlled trial

**DOI:** 10.1186/s13063-015-0809-8

**Published:** 2015-08-06

**Authors:** Roger Olofsson Bagge, Lars Ny, Charlotta All-Ericsson, Malin Sternby Eilard, Magnus Rizell, Christian Cahlin, Ulrika Stierner, Ulf Lönn, Johan Hansson, Ingrid Ljuslinder, Lotta Lundgren, Gustav Ullenhag, Jens Folke Kiilgaard, Jonas Nilsson, Per Lindnér

**Affiliations:** Department of Surgery, Institute of Clinical Sciences, Sahlgrenska Academy at University of Gothenburg, Sahlgrenska University Hospital, 413 45 Gothenburg, Sweden; Department of Oncology, Institute of Clinical Sciences, Sahlgrenska Academy at University of Gothenburg, Sahlgrenska University Hospital, Gothenburg, Sweden; St. Erik Eye Hospital, Karolinska Institutet, Stockholm, Sweden; Transplant Institute, Institute of Clinical Sciences, Sahlgrenska Academy at University of Gothenburg, Sahlgrenska University Hospital, Gothenburg, Sweden; Department of Oncology, Linköping University Hospital, Linköping, Sweden; Department of Oncology, Karolinska University Hospital, Stockholm, Sweden; Department of Oncology, Norrlands University Hospital, Umeå, Sweden; Department of Oncology, Skåne University Hospital, Lund, Sweden; Department of Radiology, Oncology and Radiation Science, Section of Oncology, Uppsala University, Uppsala, Sweden; Department of Ophthalmology, Glostrup Hospital, Copenhagen University Hospital Glostrup, Glostrup, Denmark; Sahlgrenska Cancer Center, Sahlgrenska Academy at University of Gothenburg, Sahlgrenska University Hospital, Gothenburg, Sweden

## Erratum

After publication of this work [1], we noted that we failed to include the complete list of all coauthors. The full list of authors has now been updated. The Authors’ contributions and Competing interests section modified accordingly. We are publishing this erratum to update the author list, which is as follows:

Roger Olofsson, Lars Ny, Charlotta All-Ericsson, Malin Sternby Eilard, Magnus Rizell, Christian Cahlin, Ulrika Stierner, Ulf Lönn, Johan Hansson, Ingrid Ljuslinder, Lotta Lundgren, Gustav Ullenhag, Jens Folke Kiilgaard, Jonas Nilsson and Per Lindnér.
